# Thermal experience during embryogenesis contributes to the induction of dwarfism in whitefish *Coregonus lavaretus*

**DOI:** 10.1371/journal.pone.0185384

**Published:** 2017-09-25

**Authors:** Peter Steinbacher, Josef Wanzenböck, Magdalena Brandauer, Raphael Holper, Jasmin Landertshammer, Magdalena Mayr, Christian Platzl, Walter Stoiber

**Affiliations:** 1 Division of Animal Structure and Function, Department of Cell Biology and Physiology, University of Salzburg, Salzburg, Austria; 2 Research Institute for Limnology Mondsee, University of Innsbruck, Mondsee, Austria; Universitat de Barcelona, SPAIN

## Abstract

Ecotype pairs provide well-suited model systems for study of intraspecific phenotypical diversification of animals. However, little is still known about the processes that account for the development of different forms and sizes within a species, particularly in teleosts. Here, embryos of a normal-growing ‘large’ form and a dwarf form of whitefish *Coregonus lavaretus* were incubated at two temperatures that are usually experienced at their own spawning sites (2°C for the normal and 6°C for the dwarf form). All fish were subjected to similar thermal treatment after hatching. The present data demonstrate for the first time that different thermal experience in embryonic life has lasting effects on body and muscle growth of this ecotype pair and contributes to the development of the dwarf form. Thus, juvenile fish of the regular form are much smaller and have less muscle mass when pre-hatching thermal conditions were similar to those typical for the spawning sites of the dwarf form (6°C) than when subjected to conditions of their own spawning sites (2°C). Surprisingly, fish of the dwarf form exhibit a similar pattern of response to thermal history (2°-fish much larger than 6°-fish), indicating that in their case, normal spawning site temperature (6°C) is indeed likely to act as a growth limiting factor. Results also demonstrate that the hypertrophic and hyperplastic muscle growth modes are similarly affected by thermal history. Immunolabelling experiments for Pax7, H3P and Mef2 provide evidence that the cellular mechanisms behind the increased growth rates after cold incubation in both ecotypes are increased proliferation and reduced differentiation rates of muscle precursor cells. This is of major significance to aspects of ecological and developmental biology and from the evolutionary perspective.

## Introduction

Several aquatic habitats around the world are inhabited by teleost ecotype pairs that differ in habitat utilisation, spawning behaviour and maximum size. Such ecotype pairs provide well-suited model systems for the study of intraspecific phenotypical diversification (e.g. [1–4]). Just as their North American congeners [5–7], the *C*. *lavaretus* populations of several European lakes have given rise to sympatric ecotype pairs (sometimes also referred to as ‘species’ pairs) that diverge in life style and ultimate size, usually consisting of a small ‘dwarf’ form and a larger ‘normal’ form. For ecotype pairs from Swiss lakes it has been shown that genetic similarity is higher between the dwarf and normal forms within the individual lakes than between equally-sized forms from neighbouring lakes [1,8]. Recent morphological and genetic data further suggest that such pairs represent endpoints of ‘species’ clines vitally depending on vertical temperature gradients rather than simple dichotome splits [9,10]. While some studies have shown that behavioural and physiological differences are likely to be involved in the diversification of forms [10,11], possible effects of early life experiences are as yet unknown.

Such effects, however, are highly likely as substantial evidence has accumulated over the past 15 years that thermal experience during the embryonic period is able to exert a strong, lasting influence on teleost body and muscle growth. This means that within an individual species, fish that are large at hatching as a result of their early thermal experience, may end up as small adults, and vice versa (eg. [12]). Effects of such kind have been first described in herring which were reared at different temperatures and then transferred to ambient conditions [13]. More recent studies have demonstrated that the phenomenon is also present in other teleost species. Imprinted effects of early thermal experience on muscle development have been demonstrated for Atlantic salmon *Salmo salar* [12,14–17], halibut *Hippoglossus hippoglossus* [18], haddock *Melanogrammus aeglefinus* [19], sea bass *Dicentrarchus labrax* [20], zebrafish *Danio rerio* [21], pearlfish *Rutilus meidingeri* [22] and turbot *Scophthalmus maximus* [23]. Thereby, body and muscle growth patterns have been shown to be affected in a species-specific manner, but also appear to depend upon the duration of the imprinting experiment, and the age of the specimens tested. For example, increasedrates of fast fibre formation in larvae and/or juveniles may be the result of rearing temperatures in either the lower [12,15,16] or the upper region of the species’ thermal range [18,19] during the embryonic period. Also, sea bass juveniles that originate from low temperatures have a higher and longer capacity to recruit new fast fibres when seawater temperature increases in spring [20].

Only limited information is available on whether such ‘thermal programming’ of muscle growth plays an essential role in the phenotypic diversification of teleost species—either within the normal scope of intraspecific variation or after onset of a speciation process. As to the possible induction of such temperature effects, dwarf forms of *C*. *lavaretus* from lake habitats in Central Europe are known to diverge from their large sized relatives in relation to the thermal conditions at their spawning sites. Normal forms mainly spawn at shallow sites on gravel banks of the lake’s river tributaries which are thermally labile and in winter usually cold with values down to 0.5°-2°C. By contrast, small forms are reported to prefer deeper, thermally more stable rocky littoral areas which do not cool below 4–6°C [10,24–26]. Additionally, the dwarf form is likely to spawn over an extended period from autumn to spring, while the normal form usually only spawns in late November and early December [25]. Thus, embryos of such forms are thus likely to have strongly diverging thermal histories. This may drive body and muscle growth potentials in different directions irrespective of whether the larvae eventually feed in the same superficial waters [27].

Particularly little is known as to how the thermal ‘imprint’ is implemented at the level of myogenic precursor cell recruitment. That such influence exists is highly probable from studies on small (dwarf) forms of related salmoniform species Arctic charr *Salvelinus alpinus* and Atlantic salmon. These studies provide evidence that the small condition is associated with an early cease of hyperplasia and, in consequence, with a reduced fibre number in the dominant fast muscle mass of the adults [28,29].

Based on this evidence, the present work intended to test the hypothesis whether or not the size differences of a normal-sized and a dwarf form of an ecotype pair of *C*. *lavaretus* from the Austrian lake Traunsee, are conditioned by the thermal environment at the spawning sites of the ecotypes. Knowing about this has, in our understanding, most relevant implications, not only in the evolutionary and ecological contexts (formation of ecotypes in short evolutionary times, ecotype survival under climate change conditions) but also for fisheries biology, lake/river management and aquaculture economics.

## Materials and methods

### Rearing and sampling of fish

Investigations were carried out on developmental stages of two sympatric ecotypes of *Coregonus lavaretus* (Salmonidae) with different adult size. They both occur in the Traunsee, a diluvial lake at the northern rim of the Alps in Austria (47° 52’ N, 13° 48’ O). Parent animals were caught by professional fishermen at their natural spawning sites in the lake (dwarf form) and its tributary, the Traun River (normal form) in the winter season. Parent animals of the dwarf form (15 males, 19 females) had a mean total length (TL) of 21.9 ± 1.8 cm and a mean total mass of 76.5 ± 22.2 g. Parent animals of the normal form (12 males, 15 females) were significantly larger, with a mean TL of 32.8 ± 32.7 cm and weighed 260.1 ± 127.8 g. Three batch crosses of ≥ 4 males and ≥ 5 females per ecotype were produced. Batch crosses of each ecotype were merged and transferred to Zuger glasses for rearing under controlled conditions. For thermal imprinting, batches of eggs of each ecotype were kept at two different temperatures (2°C and 6°C, ± 0.5°C each) until hatching thus forming a cold-imprinted and a warm-imprinted study group for each ecotype. Temperatures chosen are within the species’ range of thermal tolerance during embryogenesis [30] and occur at the natural spawning sites of the normal form in the river (about 2°C) and the dwarf form in the lake (5–6°C). From hatching onwards, all fish were kept under the same (season-dependent) water temperatures as prevailing in the water supplies of the Mondsee Institute. Temperatures rose steadily from 7°C in early larval life (March) to a maximum of 20°C towards the larva/juvenile transition (August), and fell to 15°C during the following autumn period. Fish were kept under natural photoperiod and fed *ad libitum*, initially with non-enriched *Artemia* nauplii, later switching to a diet of commercial fish food of appropriate particle size. Rates of water flow and recirculation were kept equal for all thermal groups at any particular phase of the experiment. Three tank replicates of each thermal group were maintained in all rearing phases. Samples of n≥7 per thermal group and method of analysis (in total ≥ 30 per thermal group) were randomly taken from the replicate tanks or each ecotype at hatching, and at 80 days post hatching (dph) in the juvenile stage. All fish were overanaesthetised with MS-222 (3-aminobenzoic acid ethyl ester, Sigma, Vienna, Austria) to minimize suffering (according to the Directive 2010/63/EU of the European Parliament and of the Council of the European Union, September 22, 2010), and body lengths were measured (n≥54 at 0 dph, and ≥40 at 80 dph) prior to fixation. Animals at 80 dph were cut into smaller pieces to allow sufficient fixative penetration.

In accordance with the regulations of the Austrian Animal Experiment Act (December 28, 2012) (Tierversuchsrechtsänderungsgesetz, part 1, section 1, §1, point 2), and with the Directive 2010/63/EU of the European Parliament and of the Council of the European Union (September 22, 2010) on the protection of animals used for scientific purposes (chapter 1, article 1, point 5a), all fish were reared according to normal agricultural practice, including provision for appropriate tank size, sufficient rate of waterflow, natural photoperiod, ad libitum food supply, and temperatures within the species’ thermal tolerance range of 1°-7°C [30]). This ensured that no pain, suffering, distress or lasting harm was inflicted on the animals, which is confirmed by the fact that mortality rates and development of aberrant fish were low and equal between rearing groups. Based on the legislative provisions above, no ethics approval and no IACUC protocol is required for the experiment performed.

### Light microscopy and morphometry

Specimens intended for light microscopic analysis and morphometry of cellularity on semithin sections (6–8 animals per thermal group and developmental stage) were immersion-fixed at 4°C using Karnovsky's paraformaldehyde-glutaraldehyde fixative [31] diluted to half-concentration with PBS. After postfixation in PBS-buffered 1% osmium tetroxide (3 h, 4°C), specimens were dehydrated in a graded series of ethanols (70–100%) and embedded into Glycid ether 100 epoxy resin (Serva, Heidelberg, Germany). Transverse semithin sections (1.5 μm) at anal level were cut on a Reichert Ultracut S microtome and mounted on glass slides. Amplification of osmium contrast for clearer visualisation of cell borders was performed using1,4-paraphenylendiamine (Merck-Schuchardt, Hohenbrunn, Germany) [32]. Results were photographed using a Reichert Polyvar microscope.

Total cross-sectional areas (csa) of the slow and fast muscle domains of one epaxial and one hypaxial trunk quadrant of each fish were digitally measured from the images. For morphometric assessment of cellularity, contours of the slow and fast muscle cells were digitally traced. In individuals at hatching, this was done for one entire epaxial quadrant without defining slow and fast muscle subzones (S1 Fig). In juveniles at 80 dph, epaxial quadrants were divided into distinct subzones according to a body-size dependent schedule. This served to detect possible local variations of hyperplastic growth in the myotome in the time after hatching. Two subzones (apical, central) in the fast muscle domain and an apical subzone in the slow muscle domain were defined. The dorsal-most 10% of the quandrant height (h) were assigned to the apical zone (characterised by stratified hyperplasia), the central third of the fast muscle’ dorso-ventral extension (midline at h/2) to the central zone (cf. [33]). Fibre size measurement in the central zone was confined to a representative transect (see [22]) (S1 Fig). Fibre numbers were always determined for the entire quadrant. All measurements were done using an adapted version of the Image J software. A few implausibly small values (< 2 μm^2^) presumed to be measuring errors were deleted from the dataset.

### Myonuclei counting

The protocol for assessment of myonuclear densities of fast muscle fibres was modified after that of our colleagues [34]. 5–10 myotomes were dissected from the anal area of fish fixed in 4% paraformaldehyde (PFA) at 4°C for 8 h. The slow fibre layer and the adhering skin were removed with fine forceps. The remaining fast muscle parts of the myotomes were incubated in 40% NaOH for 5–10 min, rinsed several times in PBS + 0,1% Tween 20, and pipetted up and down vigorously to separate individual fibres. Nuclei of muscle fibres were labelled with 10μg/ml Hoechst 33258 (Sigma, Vienna, Austria). The nuclei of 40–50 fast fibres per individual fish from 6–8 individuals per developmental stage, ecotype and thermal group were counted. Measurement of fibre lengths and diameters was done using Photoshop CS3. Data are presented as the ratio of number of myonuclei to muscle fibre volume (l r^2^ п), briefly termed nuclei/volume ratio.

### Immunolabelling

For analysis of the temperature-dependent variation of the presence of myogenic precursor cells (MPCs) in the DM and the myotome, immunolabelling experiments using paired box transcription factor 7 (Pax7+)antisera were performed on transverse cryostat sections. Freshly killed animals were coated with cryostat embedding medium (Tissue-Tek O.C.T. compound, Miles, Elkhart, USA) and cryofixed by plunging into isopentane (2-methylbutane) cooled to near its freezing point (-158°C) by liquid nitrogen. Transverse sections (10 μm) at the level of the anus were cut on a cryostat as described by [35].

Double labelling was performed to discriminate between mitotically active and differentiating MPCs subsets. Therefore, the Pax7 antibody was combined with either a marker of mitotically active cells (phospho-histone H3, H3P) or a marker of differentiating muscle cells(myogenic enhancer factor 2, MEF2). The following primary antibodies were used: monoclonal mouse anti-chicken Pax7 IgG1 (1:20; DSHB, Iowa, USA, deposited by A. Kawakami), polyclonal rabbit anti-H3P (1:100; Upstate, Lake Placid, NY, USA), and polyclonal rabbit anti-rat MEF2 (1:100; Santa Cruz Biotechnology, Santa Cruz, California, USA). Alexa 488-conjugated goat anti-rabbit (1:800) and Alexa 546-conjugated goat anti-mouse IgG1 (1:800; Invitrogen, Lofer, Austria) were applied as secondary antibodies. For immunolabelling, cryostat sections were fixed in 4% PFA (5 min), washed in PBS containing 0.1% Tween 20 (PBT) (3x3 min) and then blocked with PBT containing 2% BSA and 5% goat serum (PBT-B-N; 5 min). This was followed by primary antibody incubation (1–3 h at room temperature, or overnight at 4°C), 3x3 min washing in PBT, another blocking step in PBT-B-N (5 min), and secondary antibody incubation (30–60 min at room temperature). After washing in PBT (3x3 min), sections were counterstained with Hoechst 33258 (1 μg/ml, 3 min) to visualise nuclei, again washed in PBT (2x3 min) and then mounted with Roti^®^-Mount Fluor-Care (Roth, Austria). Photographs of the results were taken on a Reichert Polyvar microscope adapted for fluorescence microscopy.

Quantitative assessment of proliferating (Pax7+/H3P+) and differentiating (Pax7+/Mef2+) muscle precursors was carried out in the DM area immediately lateral to the slow fibres and in the lateral fast muscle area immediately medial to the slow fibres. In fish at hatching (6–16 individuals per ecotype and thermal group), these two areas were analysed in the entire epaxial and hypaxial quadrants (for epaxial quadrants exemplified in S2 Fig). In fish at 80 dph (6–7 individuals per ecotype and thermal group), evaluation was confined to individual somites/myotomes, i.e. areas delimited by two successive myosepta (cf. [22, 36]) (S2 Fig). Of each individual examined, one set of sections (mainly 5 slides with 8 sections each) was used to determine numbers of Pax7+/H3P− and Pax7+/H3P+ cells (10–20 somites/myotomes per individual, resulting in a total of about 80 evaluated somites/myotomes from 6–8 individuals per ecotype, developmental stage and thermal regime). A second set of sections was used to evaluate numbers of Pax7+/Mef2− and Pax7+/Mef2+ cells within the lateral fast muscle. Labelled cells were counted on matched images using Adobe Photoshop. Cell numbers are given per 100 μm of somite/myotome contour length (as measured on 10 μm cross sections). Note that the central areas of the myotomes (mosaic growth zone) were not included into Pax7+ cell evaluation because, with a very few exceptions, such cells were not detected at these sites (see also [33,37].

### Statistical analysis

The data were tested for equal variance and normality using Levene´s test and Kolmogorov-Smirnov test, respectively (SPSS 24, SPSS Inc., Chicago, Illinois, USA). Because the data did not satisfy the assumptions of parametric homoscedastic additive models (tests for normal distribution and homogeneity of variance failed), differences in measured variables between experimental groups were tested by nonparametric methods. Main effects and interaction effects between independent variables (temperature, group) and measured response variables were examined by nonparametric rank-based, 2-way ANOVA type tests using the R package nparLD [38]. Significance levels were set at P < 0.05.

## Results

### Fish growth

**Fig 1:**
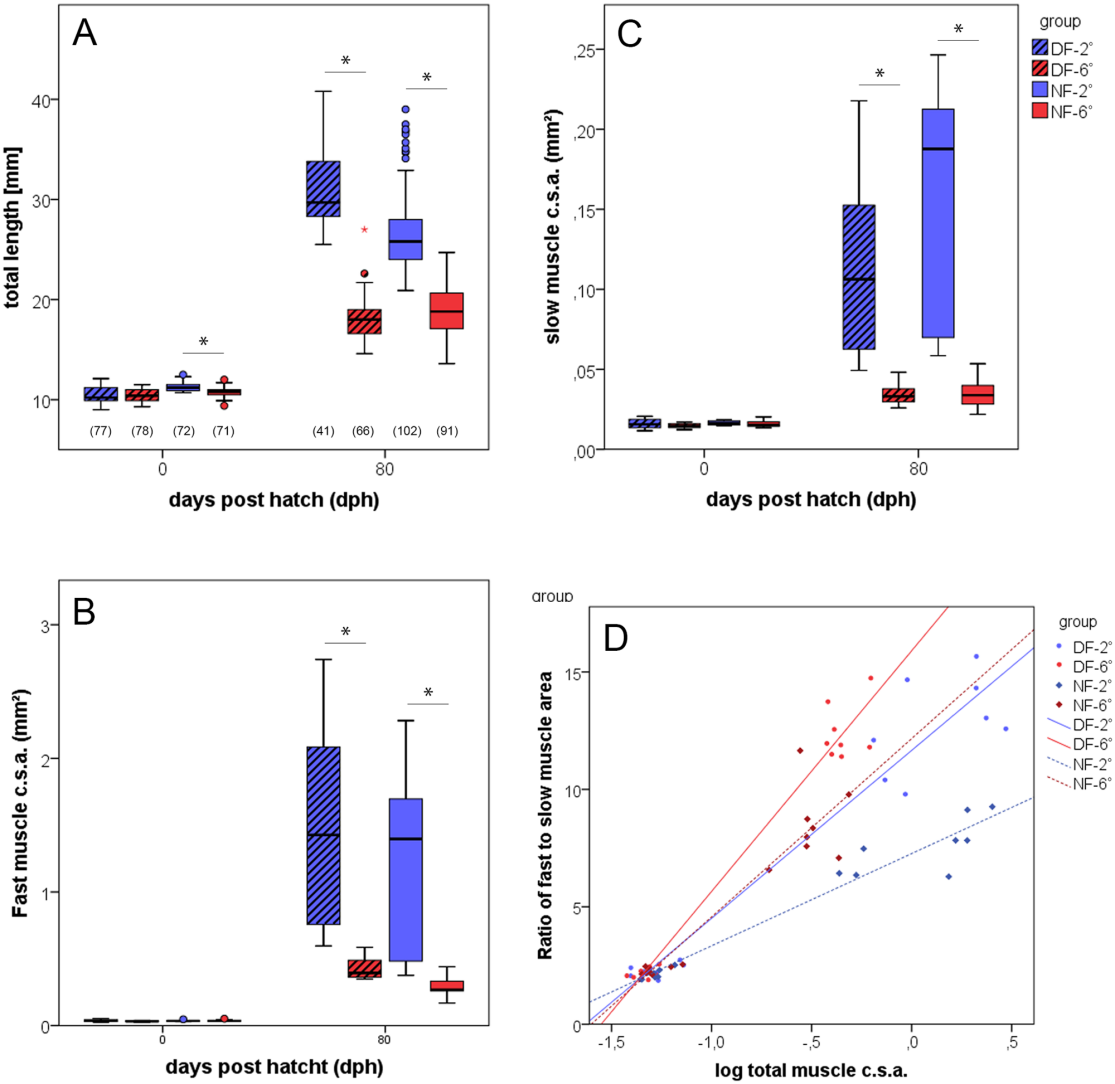
Development of body lengths and muscle mass. (A) Total body lengths of normal-sized fish (NF, open bars) and dwarf fish (DF, dashed bars) imprinted at 2 (blue) and 6°C (red) at the end of the imprinting period (0 dph) and in the juvenile stage (80 dph). Values at bottom of bars provide number of individuals included in length measurement. Total fast (B) and slow (C) muscle csa in one half of the trunk (8 individuals per thermal group of each ecotype); whiskers indicate s.e., significant differences are assigned at p≤0.05 (*). (D) Correlation of slow muscle relative proportion (fast-to-slow muscle ratio) with fish size as given by total muscle csa; regression line equations: NF-2: y = 7.3x + 3.9 (r^2^ = 0.87), NF-6: y = 12.2x + 7.6 (r^2^ = 0.95), DF-2: y = 11.7x + 7.2 (r^2^ = 0.94), DF-6: y = 15.9x + 10.3 (r^2^ = 0.97).

Fish reared at warmer temperatures developed faster than those kept under colder thermal conditions throughout the embryonic period. Thus, embryos hatched at 64 and 122 days post fertilisation (normal form, NF), and 57 and 109 days post fertilization (dwarf form, DF) in the 6° and 2°C rearing groups, respectively. The combined mean body lengths at this stage were significantly different between the two forms, being 11.0 ± 0.5 mm for the normal form and 10.5 ± 0.7 mm for the dwarf form (P<0.001). Comparison between the thermal groups of each form further revealed that the temperature, at which fish were kept during the ‘imprinting’ period until hatching only significantly influenced the growth of the normal form. If kept at 2°C, fish of this form were significantly larger than their congeners reared at 6°C (P<0.001) (Fig 1A). Under the ambient thermal conditions after hatching, cold-imprinted fish of the normal form exhibited a more pronounced growth than the warm-imprinted fish of this form, thus being clearly larger than the latter at 80 dph (mean TL at 26.8 ± 0.4 mm and 18.9 ± 0.3 mm, respectively). A similar size difference at 80 dph was observed between cold- and warm-imprinted fish of the dwarf form (mean TL 31.0 ± 0.5 mm and 18.0 ± 0.2 mm, respectively). Body size comparison between the fish of the two ecotypes at 80 dph without considering the imprinting regime did not reveal a significant difference.

### Muscle cross section and cellularity

In fish at the hatching stage, the total cross sectional area (csa) of the fast muscle domain within one half of the trunk did not significantly diverge between thermal groups and ecotypes (Fig 1B). By contrast, at 80 dph, cold-imprinted fish had larger fast muscle csa than the warm-imprinted individuals in both ecotypes (P<0.001 each). No such differences occurred between fish of the normal and dwarf form imprinted at the same thermal conditions. Similar to the results in fast muscle, slow muscle csa at hatching showed no significant differences between any of the groups (Fig 1C), and again, the situation changed at 80 dph, showing a higher slow muscle csa in the cold-imprinted fish of both ecotypes (P<0.001 each). Plotting the ratio of fast-to-slow muscle csa against total muscle csa demonstrates that cold-reared fish of both ecotypes have lower fast-to-slow muscle ratios than their warm-bred congeners. This is in agreement with the previous finding that cold acclimation favour slow muscle development over fast muscle development [35].

**Fig 2:**
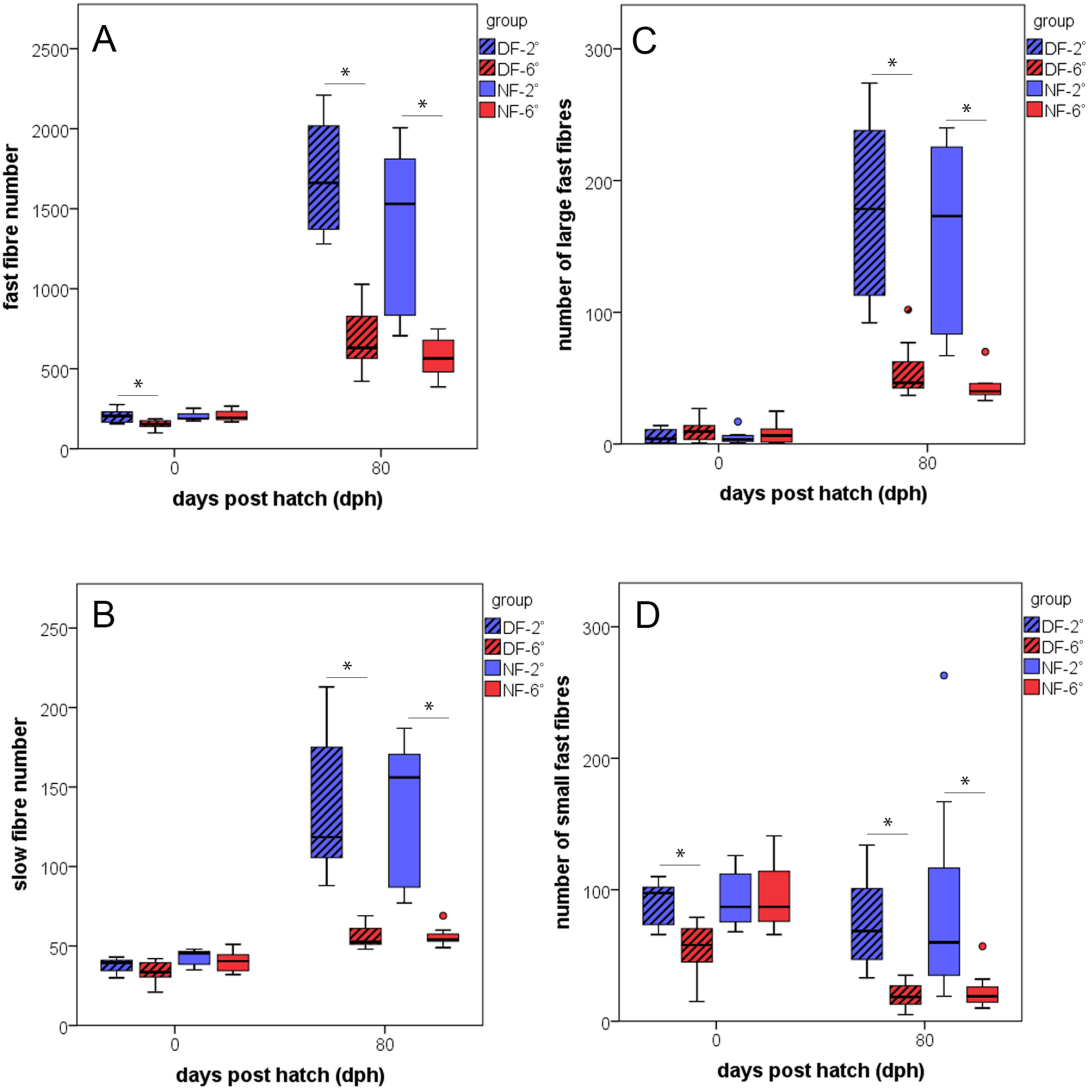
Development of slow and fast muscle fibre numbers. Relationship between fibre number in one epaxial trunk quadrant of normal-sized fish (NF, open bars) and dwarf fish (DF, dashed bars) imprinted at 2° (blue) and 6°C (red) and developmental time (8 individuals per thermal group of each ecotype). (A) Fast fibres, (B) slow fibres, (C) fast fibres > 200 μm^2^, (D) fast fibres ≤ 50 μm^2^; whiskers indicate s.e., differences between thermal groups significant at p≤0.05 (*).

In regard to fibre numbers at hatching, the cold-imprinted fish of the dwarf form contained significantly more fast fibres than those of all other study groups (P<0.001) (Fig 2A). By contrast, there were no significant differences in slow fibre numbers between thermal groups and ecotypes at this stage (Fig 2B). More prominent differences in fibre numbers were observed at 80 dph. At this time, the cold-imprinted individuals of both forms exhibited significantly elevated numbers of slow and fast fibres as compared to warm-imprinted individuals (P<0.001 each). The increase in fast fibre number over the investigated period (from hatching to 80 dph), taken as a measure of hyperplastic growth intensity, is 6.9-fold and 8.3-fold in cold-imprinted fish of the normal and dwarf form, respectively (corresponding to an increase of 14.8 and 18.7 fibres/day, respectively), but only 2.8- and 4.5-fold in the warm-imprinted fish of the two forms (corresponding to 4.6 and 6.7 fibres/day, respectively). Slow fibre numbers exhibited similar differences related to imprinting temperature, with a 3.2- and 3.6-fold increase in cold-imprinted fish, but only an 1.4- and 1.7-fold increase in warm imprinted fish of the normal and the dwarf form, respectively.

Numbers of large fast muscle fibres > 200 μm^2^ within the epaxial quadrant of the trunk were measured to determine muscle fibre hypertrophy. At hatching, there were no marked differences in this variable between any of the study groups (Fig 2C). This was changed by enhanced fast fibre growth during the larval and early juvenile period attendant to cold imprinting. Thus, at 80 dph, the cold-imprinted fish of both ecotypes contained significantly more large fast fibres than their warm-imprinted counterparts (P<0.001 each).

Small fast fibres ≤ 50 μm^2^ (which were regarded as newly formed) were counted as a means to specifically examine how thermal imprinting influences stratified and mosaic hyperplastic growth. At hatching, thermal groups of the dwarf form diverge significantly in this variable, with significantly more small fibres in the cold imprinted fish (P<0.001) (Fig 2D); thermal groups of the normal form do not exhibit this difference. However, when comparison is made between ecotypes without considering thermal groups, newly hatched fish of the normal type contain more small fibres than those of the dwarf type. By contrast, more small fast fibres were present in the normal-sized forms than in the dwarf fish (P = 0.04). In the juvenile fish at 80 dph, analysis of small fast fibre numbers was restricted to the apical and central zones of the analysed quadrants, which are known to be representative of stratified and mosaic growth, respectively. Results demonstrate that cold-imprinted fish at 80 dph, generally possess more small fibres than warm-imprinted fish, irrespective of ecotype. This is particularly clear for the central zone of the myotome (difference significant at P<0.001 in each ecotype). A slightly different situation is encountered in the apical zone, for which a significant difference in small fast fibre numbers between cold- and warm-imprinted fish was only found in the dwarf form (P<0.001), while there is only a trend towards more such fibres in cold-imprinted fish of the normal form (P = 0.11). No significant differences of such kind were detected for small slow fibre numbers (not shown).

**Fig 3:**
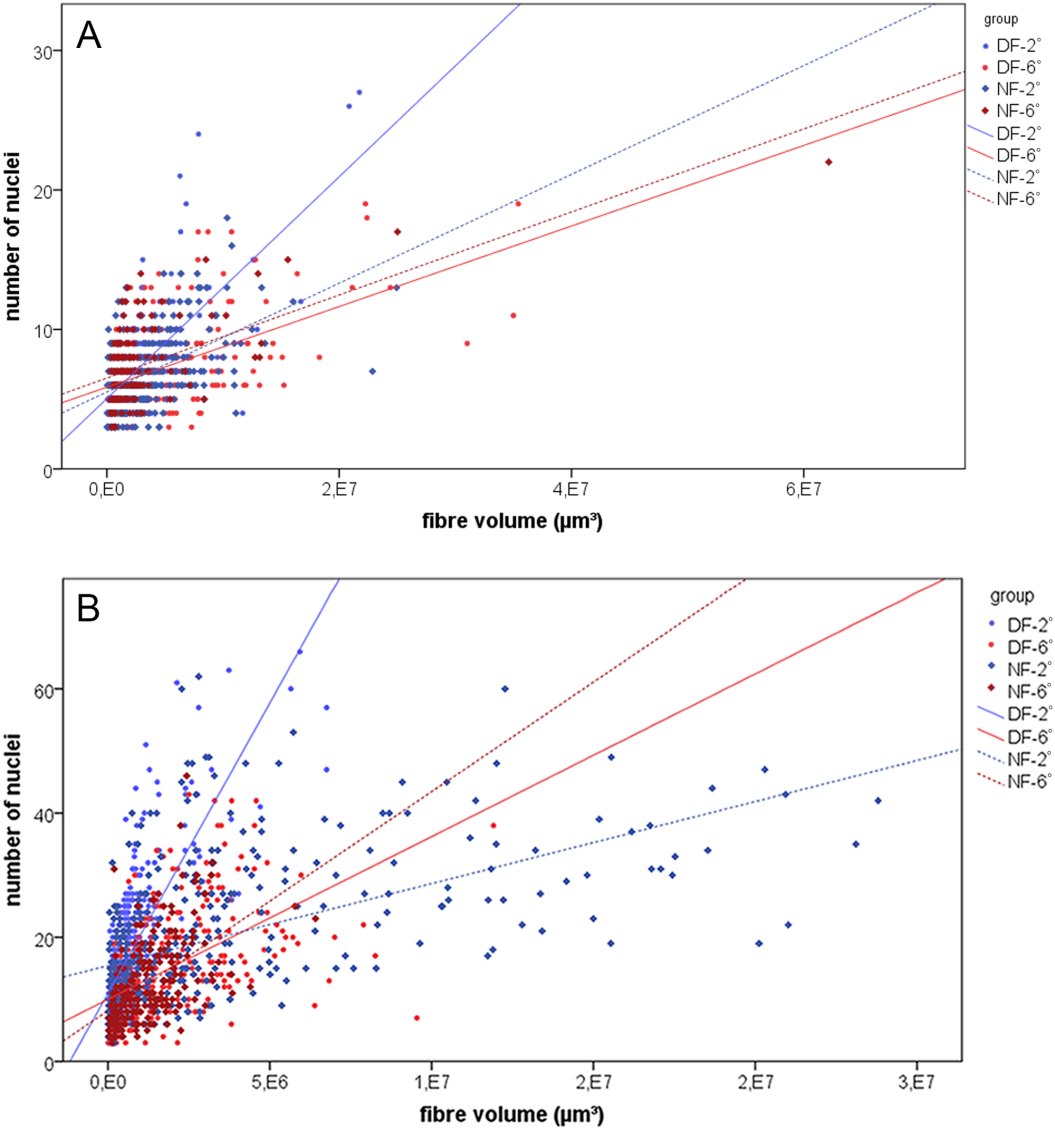
Relationship of nuclei to fibre volume of isolated fast fibres. Myonuclear densities at hatching (A) and 80 dph (B). Regression line equations: (A) NF-2: y = 3.9e^-6^x + 5.5 (r^2^ = 0.25) (356 fibres from 6 individuals), NF-6: y = 3.0e^-6^x + 6.5 (r^2^ = 0.38) (126 fibres from 6 individuals), DF-2: y = 8.0e^-6^x + 5.1 (r^2^ = 0.48) (303 fibres from 6 individuals), DF-6: y = 2.9e^-6^x + 5.9 (r^2^ = 0.26) (197 fibres from 6 individuals); (B): NF-2: y = 1.3e^-6^x + 15.4 (r^2^ = 0.28) (376 fibres from 8 individuals), NF-6: y = 3.5e^-6^x + 8.2 (r^2^ = 0.27) (236 fibres from 7 individuals), DF-2: y = 9.4e^-6^x + 11.0 (r^2^ = 0.59) (552 fibres from 8 individuals), DF-6 y = 2.6e^-6^x + 10.0 (r^2^ = 0.29) (332 fibres from 8 individuals).

Evaluation of myonuclear density demonstrates that at hatching, the fast fibres of warm-imprinted fish had a lower myonuclei/volume ratio (i.e. larger nuclear domains) than those of cold-imprinted fish in both ecotypes (Fig 3A). In the normal form, this situation became inverted during the post-imprinting period, resulting in the lowest fast fibre nuclei/volume ratio of all study groups in the cold-imprinted fish at 80 dph (Fig 3B). By contrast, no such change occurred in the dwarf form, in which the fast fibres of the warm-imprinted fish maintained a lower nuclei/volume ratio until this point in development.

### Muscle precursor cells

**Fig 4:**
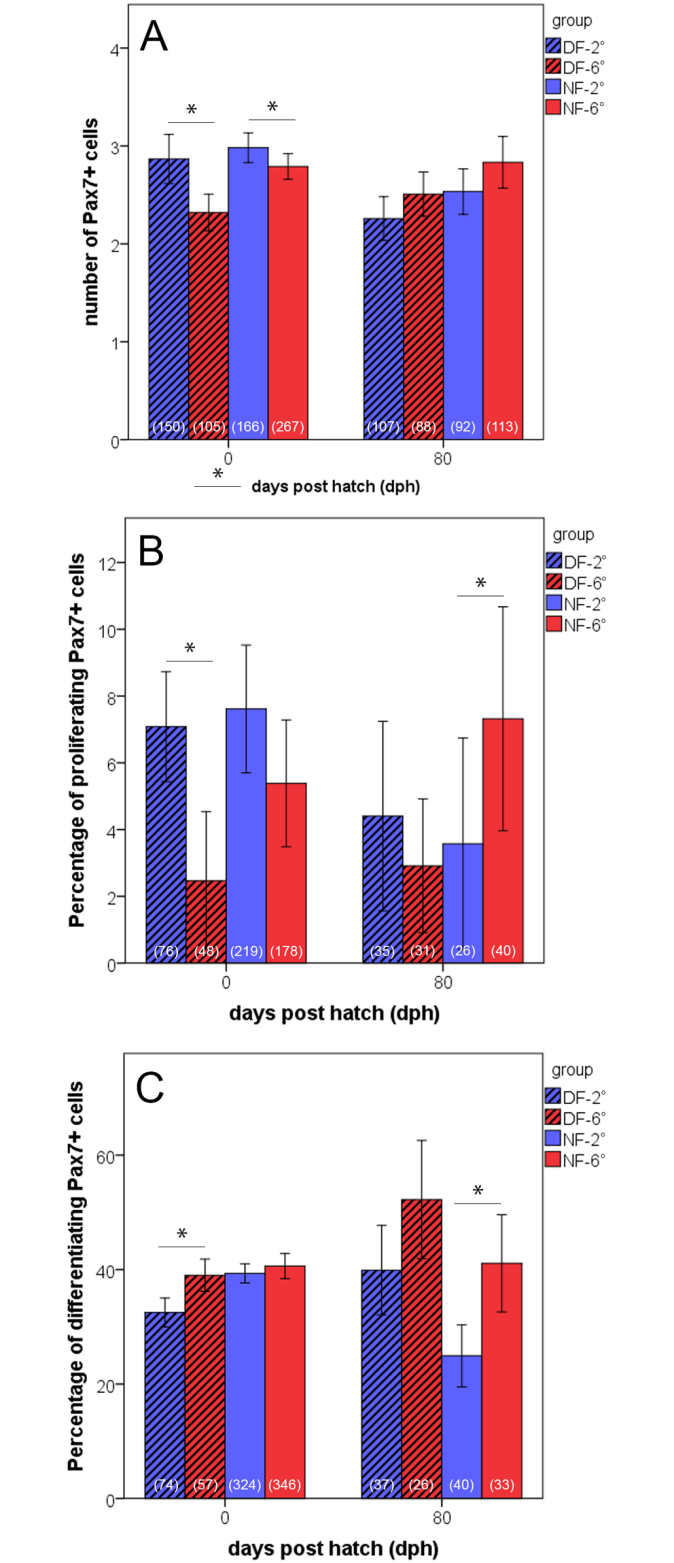
Quantification of muscle precursor cells (MPCs). Numbers of labelled cells (means + s.e.) in double-immunostained 10 μm myotomal cross-sections of fish of the normal-sized form (NF, open bars) and dwarf fish (DF, dashed bars) imprinted at 2° (blue) and 6°C (red) at hatching and at 80 dph. Values at bottom of bars provide total numbers of evaluated quadrants (fish at hatching) and somite/myotome areas delimited by 2 successive myosepta (fish at 80 dph), respectively. Data at hatching derived from 16 2°-fish and 6°- fish each in the normal form, and from 8 2°-fish and 6°-fish each in the dwarf form; data at 80 dph derived from 7 individuals in all four groups. (A) Total numbers of Pax7+ cells per 100 μm distance within the DM and the lateral fast muscle. (B,C) Percentages of Pax7+ cells that have entered proliferation (Pax7+/H3P+) (B) or differentiation (Pax7+/Mgn+) (C). (*) Intergroup differences significant at p≤0.05.

Immunohistochemical tracing of MPCs served to reveal how proliferation and differentiation behaviour of precursor cells correlates with those of the observed temperature-dependent modulation of muscle cellularity. MPCs were stained using Pax7 antiserum, which is a reliable and widely applied marker of myogenic precursor cells in vertebrates (eg. [39]). Immunolabelling data are given in relative numbers (labelled Pax7+ cells per 100 μm of myotome cross sectional length). Results show that at hatching, Pax7+ MPCs nearly entirely occurred within the dermomyotome (DM) which by that time still constitutes an epithelium at the lateral surface of the myotomes’ slow muscle layer. Most Pax7+ MPCs were detected in the DM of cold-reared specimens of both ecotypes (P<0.001, each) (Fig 4A). Co-staining for H3P demonstrates that the DM of the cold-incubated fish of the dwarf form also harbours the largest fraction of proliferating (Pax7+/H3P+) MPCs (7.1% as compared to 2.5% recorded in the warm-incubated fish) (Fig 4B). Cold-incubated fish of the normal form also had higher numbers of such cells than their warm-incubated counterparts (7.6% vs. 5.4%), although the difference is not statistically significant (P = 0.08) (Fig 4B). Regarding the percentage of differentiating MPCs at hatching (i.e. at the end of the imprinting period), a clear temperature effect was only found in the dwarf form, in which the cold-incubated fish contained lower numbers of differentiating MPCs than the warm-incubated (32.5% and 39.0% of all MPCs in 2°- and 6°-dwarf fish, respectively) (P<0.001) (Fig 4C). In contrast to hatching stage, no significant differences in numbers of Pax7+ MPCs were present at 80 dph between any of the study groups (Fig 4A). The only difference found at this stage was in regard to the relative percentage of mitotically active MPCs between the cold- and warm-imprinted fish of the normal form (Fig 4B). In the warm-imprinted fish of this ecotype, 6.6% of all Pax7+ cells were double-labelled for H3P, compared to only 2.7% of in the cold-imprinted fish (P<0.001). The highest amounts of differentiating (Mef2+) MPCs were present in the warm-imprinted specimens of both ecotypes (Fig 4C). A particularly clear difference in this respect was found in the normal form, with 41.0% and 24.7% of all Pax7+ cells being also positive for Mef2 in the warm- and cold-inprinted fish, respectively (P<0.001). A similar trend was also observed in the dwarf form (52.2% and 39.7% in warm- and cold-imprinted fish, respectively; P = 0.14).

## Discussion

The present study demonstrates clearly that thermal experience during early ontogeny exerts a long-lasting influence on body and muscle growth in whitefish and, even more important, that fish sharing the same thermal history have similar body and muscle growth, irrespective of whether they belong to the normal form or to the dwarf form of the ecotype pair. The first line of evidence in support of this is provided by the body size data. While there is no difference in body length and muscle mass (csa) between the two thermal groups of each ecotype at hatching, fish imprinted at 2°C are in both ecotypes significantly larger and have more muscle mass than fish imprinted at 6°C at 80 dph (Fig 1A–1C). This result is in remarkable agreement with, and further supports, recent morphological and genetic data interpreted in the way that the evolutionary diversification of coregonid fishes generates endpoints of ‘species’ clines vitally depending upon the vertical gradient in depth and water temperature rather than simple dichotome splits [9,10]. Further work in this direction has already suggested that metabolic and behavioural adaptations to different thermal microhabitats along the lake´s temperature-depth gradient aid the evolutionary branching of the ancestral population into two distinct phenotypes [40]. The present data further substantiates this interpretation by demonstrating that the phenotypic differences within the investigated ecotype pair are substantially influenced by different thermal environments during embryogenesis in that thermal experience in early life exerts an imprinting effect on the cellular growth mechanisms in post-embryonic life.

This conforms with, and adds to previous evidence of a similar type of imprinting effect from a variety of teleost species. As in the present experiment, in all such cases, egg incubation under a warm regime led to larger fish at the end of the embryonic period than incubation under a cold regime, while it happened very often that the cold-bred fish underwent a significant catch-up growth in the period afterwards (eg. [20,23,41]). In Atlantic salmon [12,14,16] and Atlantic herring [42], such fish were the largest at the end of the experiment. By contrast, fish of other teleost species, such as halibut, haddock and zebrafish were shown to have reduced post-embryonic muscle growth in response to cold rearing during the embryonic period [18,19,21]. This demonstrates that long-term teleost growth responses to embryonic thermal conditions are not unidirectional and likely dependent upon additional factors. A more complex and plastic nature of thermal history associated growth responses is supported by the finding in zebrafish that lasting influence may not only be exerted on final size, but also on the morphology of various body parts, such as fins, gill covers and lower jaws [43].

Imprinting effects established for body length and muscle csa are clearly reflected at the cellularity level. Also here, effects were hardly visible at the end of the embryonic period, but became most distinct during the larval and juvenile period until 80 dph. Thus, the hatching stage was characterized by no or only minor differences in fast and slow fibre numbers between thermal groups in both ecotypes, while the cold-imprinted fish surpassed the warm-imprinted fish in all relevant variables afterwards [Fig 2A and 2B]. The analyses of numbers of small (≤ 50 μm^2^) and large (> 200 μm^2^) slow and fast fibres, and of myonuclear densities identify increased rates of hyperplastic and hypertrophic growth in the cold-imprinted fish as a main reason for the diverging development in the post-embryonic time. Also in this case, effects were hardly visible at hatching but most prominent in the juvenile period [Figs 2C, 2D, 3A and 3B]. An aspect worth to be considered in more detail is that fast fibre hypertrophy in the cold-imprinted fish comes along with larger nuclear domains, as indicated by reduced myonuclear densities [Fig 3]. Two interpretations remain possible as to this observation: (i) imprinting at 2°C favours increase in cell volume without requiring uptake of more nuclei from MPCs; (ii) cold imprinting reduces the availability of MPC derived nuclei for fusion with growing fibres thus accounting for early nuclear domain expansion. The findings that numbers of large fibres do not diverge between temperature groups at hatching while the MPCs of cold-reared fish have higher proliferation rates but lower differentiation rates [Figs 2C, 4B and 4C] are clearly in support of the latter hypothesis. The response of nuclear domains established in whitefish bears a striking resemblance to those found in the pearlfish *Rutilus meidingeri*, a large-growing Central European cyprinid, in which cold-imprinted fish also had higher nuclear densities at hatching but reduced densities in the juvenile period [22]. There are also further similarities in imprinting responses between pearlfish and whitefish, especially with regard to the fact that slow muscle responds very much like fast muscle in both overall mass and cellularity, and in variation of the relative proportion of the slow muscle compartment (fast/slow-ratio).

In contrast to the whitefish, however, cold-imprinted pearlfish had a lower posthatching body and muscle growth potential indicating that a high nuclear density of the muscle fibres at hatching does not necessarily reflect the subsequent hypertrophic and/or hyperplastic growth potential of the muscle. This fits with the wider observation that imprinting effects in whitefish do not in all aspects correspond to those resulting from similar experimentation in other temperate sea- and freshwater species, eg. Atlantic salmon [44], herring [13], and haddock [19]. While in these species, just as in the pearlfish, cold-imprinting favoured hypertrophy over hyperplasia, and *vice versa* warm-imprinting hyperplasia over hypertrophy, there were no such preferential responses in whitefish. The reason for this phenomenon remains to be clarified.

At the level of molecular regulation, the results from immunostaining for Pax7, H3P and Mef2 suggest that thermally induced shift of MPC behaviour is a major driving force behind the observed effects on body growth and muscle cellularity. The relatively higher amounts of proliferating Pax7+/H3P+ cells in the cold imprinted-fish of both ecotypes at hatching, and to a lower extent still at 80 dph, together with the relatively higher numbers of differentiating Mef2+ cells in the warm-imprinted fish [Fig 4B and 4C], provide strong indication that temperature modulates the proliferation/differentiation balance of the MPCs formed during the embryonic period, leading to an increased accumulation of MPCs under cold conditions. The thus generated larger pool of MPCs appears to be the cellular basis of the increased hypertrophic and hyperplastic posthatching muscle growth of the cold imprinted fish, finally resulting in larger juveniles. This is very similar to what has been suggested to occur in the pearlfish [22], although with the difference that the pearlfish study, making comparison between three thermal regimes within the species’ tolerance range (13° vs. 8.5° and 16°C), found that not the lowest, but the intermediate temperature led to the highest MPC numbers at hatching. This was interpreted in the way that the size of the embryonic MPC pool has a species-specific thermal optimum rather that it would simply decrease along a cold-warm gradient [22]. The interpretation is supported by evidence from two studies of Atlantic salmon that densities of MPCs positive for the development-related receptor tyrosine kinase c-met+ MPCs in juvenile fast muscle are highest when the embryos were imprinted within an ‘optimum thermal range’, while colder conditions and heating experiments led to reduced densities [14,15]. Based on the present data, it may be supposed that the MPC pool optimum of the two whitefish ecotypes is in each case close to 2°C.

More recently, molecular data from the Senegalese sole (*Solea senegalensis*) have suggested an epigenetic mechanism accounting for the temperature-dependent plasticity of teleost muscle growth. It was found that rearing temperature influences the methylation of the promoter of myogenin, an important transcription factor that regulates MPC differentiation. Increased methylation under cold conditions caused a downregulation of myogenin expression [45]. Although the investigated whitefish present an inverted situation compared to the sole, with increased muscle growth after cold incubation, it may be hypothesised that similar epigenetic processes are the basis for the temperature-dependency of muscle growth in these fish. Specifically MPC proliferation rates, according to the present findings a key factor driving phenotypic diversification in this species, may be regulated by temperature mediated influence on the methylation degrees of growth factor genes such as IGF or FoxK1. That the expression of such factors reacts to temperature has already been shown in teleosts, e.g. in pufferfish *Takifugu rubripes* and carp *Cyprinus carpio* [46,47].

Taken together, the findings of the present work demonstrate that both the normal-sized and the dwarf form of *C*. *lavaretus* have equal growth potentials and the exploitation of this potential depends upon thermal experience during early (embryonic) life. Thus, fish of both forms are clearly best conditioned for post-hatching growth if the embryos experience low temperatures, independent of the temperatures at their natural spawning sites. Or, when reasoned *a contrario*, the results indicate that spawning site temperature is a relevant factor in body size diversification between the two whitefish ecotypes. This pattern of response to cold imprinting observed in whitefish is remarkably similar to that established for two populations of Atlantic salmon with different spawning behaviour [14]. Irrespective of whether fish were upland spawners adapted to lower temperature or lowland spawners adapted to higher temperatures, cold-rearing led to individuals with larger muscle csa and higher numbers of slow and fast muscle fibres at hatching and at first feeding. Observations made by [28] when comparing effects of different (colder/warmer) rearing temperatures on embryos of a large benthic and a dwarf benthic morph of Arctic charr are also in principle in agreement with the present results. An enhanced growth potential in response to low embryonic temperature is indicated by that numbers of fast fibres in cold-reared fry of the dwarf morph do not significantly diverge from those counted in warmer reared individuals of the large morph, although the latter possess more muscle fibres as adults. However, further research will be required to examine whether additional experiences during early life stages that are known to affect fish growth (e.g. oxygen availability, ammonia and nitrate level, density) also aid in the segregation of teleost ecotype pairs, and to clarify the molecular details of how exactly rearing temperature interferes with stem cell behaviour. Besides strong implications in the phylogenetic and developmental context, the present data are also important in the climatic context as with increasing water temperatures at the spawning sites body and muscle growth of such fish will decrease.

## Supporting information

S1 Fig Images illustrating contour tracing of slow fibres (red) and fast fibres (black). Epaxial quadrants of fish at hatching (A) and at 80 dph (B). Zonal subdivision at 80 dph (AZ apical zone, CZ central zone) is dependent of myotome size (details provided in the Methods section). FF fast fibres, h/w height/width of quadrant, ms myoseptum, nc notochord, SF slow fibres, spc spinal cord. Scale bars: A 50 μm, B 100 μm.
(TIF)

S2 Fig Images of sections immunostained for Pax7 (red) illustrating MPC assessment in the myotome. Dorsal is to the top. (A) Newly hatched 2°-fish of dwarf form, section co-stained for H3P (green) to test for mitotically active Pax7+ cells (white arrowhead). All Pax7+ cells are exclusively located in the area of the previous DM. (B) Newly hatched 6°-fish of normal sized-form, section co-stained for MEF2 (green) to test for Pax7+ cells entering myogenic differentiation (white arrowheads). (C) 6°-fish of dwarf form at 80 dph, section co-labelled for H3P (green). Pax7+ cells and mitotically active Pax7+/H3P+ cells generally occur in both the area of the previous DM and in the lateral fast muscle (white arrowhead indicates Pax7+/H3P+ cell). (D) 2°-fish of normal-sized form at 80 dph, section co-stained for MEF2 (green). White arrowheads indicate double-labelled cells. Nuclei are counterstained with Hoechst 33258. FF fast fibres, ms myoseptum, nc notochord, SF slow fibres. Scale bars: 100 μm.
(TIF)

## Abbreviations

csa: cross sectional area
DF: dwarf form
DM: dermomyotome
dph: days post hatching
H3P: phosphohistone H3
Mef2: myocyte enhancer factor 2
MPC: muscle precursor cell
NF: normal-sized form
Pax7: paired box transcription factor 7
TL: total length

## References

[1] Douglas MR, Brunner PC, Bernatchez L. Do assemblages of *Coregonus* (Teleostei: Salmoniformes) in the Central Alpine region of Europe represent species flocks? Mol Ecol. 1999; 8: 589–603.

[2] Bernatchez L. Ecological theory of adaptive radiation. An empirical assessment from Coregonine fishes (Salmoniformes) In: Hendry AP, Stearns SC, editors. Evolution Illuminated. Oxford University Press, Oxford; 2004 pp. 175–207.

[3] Kahilainen K, Ostbye K. Morphological differentiation and resource polymorphism in three sympatric whitefish *Coregonus lavaretus* (L.) forms in a subarctic lake. J Fish Biol. 2006; 6: 63–79.

[4] Hudson AG, Vonlanthen P, Müller R, Seehausen O. Review: the geography of speciation and adaptive radiation in Coregonines. Arch Hydrobiol. 2007; 60: 111–146.

[5] Rogers SM, Bernatchez L. The genetic architecture of ecological speciation and the association with signatures of selection in natural lake whitefish (*Coregonus* sp. Salmonidae) species pairs. Mol Biol Evol. 2007; 24: 1423–1438. doi: 10.1093/molbev/msm066 17404398 10.1093/molbev/msm066

[6] Rogers SM, Isabel N, Bernatchez L. Linkage maps of the dwarf and normal lake whitefish (*Coregonus clupeaformis*) species complex and their hybrids reveal the genetic architecture of population divergence. Genetics 2007; 175: 375–398. doi: 10.1534/genetics.106.061457 17110497 10.1534/genetics.106.061457 PMC1774998

[7] St-Cyr J, Derome N, Bernatchez L. The transcriptomics of life-history trade-offs in whitefish species pairs (*Coregonus* sp.). Mol Ecol. 2008; 17: 1850–1870. doi: 10.1111/j.1365-294X.2008.03696.x 18312278 10.1111/j.1365-294X.2008.03696.x

[8] Douglas MR, Brunner PC, Douglas ME. Evolutionary homoplasy among species flocks of Central Alpine *Coregonus* (Teleostei: Salmoniformes). Copeia 2005; 2: 347–358.

[9] Ohlberger J, Mehner T, Staaks G, Hölker F. Temperature-related physiological adaptations promote ecological divergence in a sympatric species pair of temperate freshwater fish, *Coregonus* spp. Funct Ecol. 2008; 22: 501–508.

[10] Vonlanthen P, Roy D, Hudson AG, Largiadèr CR, Bittner D, Seehausen O. Divergence along a steep ecologoical gradient in lake whitefish (*Coregonus* sp.). J Evol Biol. 2009; 22: 498–514. doi: 10.1111/j.1420-9101.2008.01670.x 19170819 10.1111/j.1420-9101.2008.01670.x

[11] Landry L, Vincent WF, Bernatchez L. Parallel evolution of lake whitefish dwarf ecotypes in association with limnological features of their adaptive landscape. J Evol Biol. 20: 971–984. doi: 10.1111/j.1420-9101.2007.01304.x 17465908 10.1111/j.1420-9101.2007.01304.x

[12] Albokhadaim I, Hammond CL, Ashton C, Simbi BH, Bayol S, Farrington S, et al Larval programming of post-hatch muscle growth and activity in Atlantic salmon (*Salmo salar)*. J Exp Biol. 2007; 210: 1735–1741. doi: 10.1242/jeb.003194 17488936 10.1242/jeb.003194

[13] Johnston IA, Cole NJ, Abercromby M, Vieira VLA. Embryonic temperature modulates muscle growth characteristics in larval and juvenile herring. J Exp Biol. 1998; 201: 623–646. 9450973 10.1242/jeb.201.5.623

[14] Johnston IA, McLay HA, Abercromby M, Robins D. Early thermal experience has different effects on growth and muscle fibre recruitment in spring- and autumn-running Atlantic salmon populations. J Exp Biol. 2000; 203: 2553–2564. 10933999 10.1242/jeb.203.17.2553

[15] Johnston IA, Manthri S, Alderson R, Smart A, Campbell P, Nickell D, et al Freshwater environment affects growth rate and muscle fibre recruitment in seawater stages of Atlantic salmon (*Salmo salar* L.). J Exp Biol. 2003; 206: 1337–1351. 12624169 10.1242/jeb.00262

[16] Macqueen DJ, Robb DHF, Olsen T, Melstveit L, Paxton CGM, Johnston IA. Temperature until the 'eyed stage' of embryogenesis programmes the growth trajectory and muscle phenotype of adult Atlantic salmon. Biol Lett. 2008; 4: 294–298. doi: 10.1098/rsbl.2007.0620 18348956 10.1098/rsbl.2007.0620 PMC2610038

[17] Côte J, Roussel JM, Le Cam S, Guillaume F, Evanno G. Adaptive divergence in embryonic thermal plasticity among Atlantic salmon populations. J Evol Biol. 2016; 29: 1593–1601. doi: 10.1111/jeb.12896 27177256 10.1111/jeb.12896

[18] Galloway TF, Kjorsvik E, Kryvi H. Muscle growth in yolk-sac larvae of the Atlantic halibut as influenced by temperature in the egg and yolk-sac stage. J Fish Biol. 1999; 55: 26–43.

[19] Martell DJ, Kieffer JD. Persistent effects of incubation temperature on muscle development in larval haddoch (*Melanogrammus aeglefinus* L.). J Exp Biol. 2007; 210: 1170–1182. doi: 10.1242/jeb.002188 17371916 10.1242/jeb.002188

[20] Alami-Durante H, Olive N, Rouel M. Early thermal history significantly affects the seasonal hyperplastic process occurring in the myotomal white muscle of *Dicentrarchus labrax* juveniles. Cell Tissue Res. 2007; 327: 553–570. doi: 10.1007/s00441-006-0321-2 17036227 10.1007/s00441-006-0321-2

[21] Johnston IA, Lee HT, Macqueen DJ, Paranthaman K, Kawashima C, Anwar A, et al Embryonic temperature affects muscle fibre recruitment in adult zebrafish: genome-wide changes in gene and microRNA expression associated with the transition from hyperplastic to hypertrophic growth phenotypes. J Exp Biol. 2009; 212: 1781–1793. doi: 10.1242/jeb.029918 19482995 10.1242/jeb.029918

[22] Steinbacher P, Marschallinger J, Obermayer A, Neuhofer A, Sänger AM, Stoiber W. Temperature-dependent modification of muscle precursor cell behaviour is an underlying reason for lasting effects on muscle cellularity and body growth of teleost fish. J Exp Biol. 214: 1791–1801. doi: 10.1242/jeb.050096 21562165 10.1242/jeb.050096 PMC3108887

[23] Ayala MD, Martínez JM, Hernández-Urcera J, Cal R. Effect of the early temperature on the growth of larvae and postlarvae turbot, *Scophthalmus maximus* L.: muscle structural and ultrastructural study. Fish Physiol Biochem. 2016; 42: 1027–1042. doi: 10.1007/s10695-015-0194-y 26762321 10.1007/s10695-015-0194-y

[24] Steinmann P Monographie der schweizerischen Koregonen. Beitrag zum Problem der Entstehung neuer Arten. Schweiz Zeits Hydrol. 1950; 12: 340–491.

[25] Hamann H. Beiträge zur Biologie und Ermittlungen zu den Fischereiverhältnissen des Traunsees 1952–1953. Report. Biologische Station für Fischereiwesen, Linz/Donau; 1954.

[26] Wanzenböck J, Gassner H, Lahnsteiner B, Hassan Y, Hauseder G, Doblander C, et al Ecological integrity assessment of lakes using fish communities: An example from Lake Traunsee exposed to intensive fishing and to effluents from soda industry. Water Air Soil Poll. 2002; 2: 227–248.

[27] Lahnsteiner B, Wanzenböck J. Variability in the spatio-temporal distribution of larval European whitefish (*Coregonus lavaretus* (L.)) in two Austrian lakes. Ann Zool Fennici 2004; 41: 75–83.

[28] Johnston IA, Abercromby M, Vieira VLA, Sigursteindóttir RJ, Kristjánsson BK, Sibthorpe D, et al Rapid evolution of muscle fibre number in post-glacial populations of arctic charr *Salvelinus alpinus*. J Exp Biol. 2004; 207: 4343–4360. doi: 10.1242/jeb.01292 15557021 10.1242/jeb.01292

[29] Johnston IA, Abercromby M, Andersen O. Loss of muscle fibres in a landlocked dwarf Atlantic salmon population. Biol Lett. 2005; 1: 419–422. doi: 10.1098/rsbl.2005.0377 17148222 10.1098/rsbl.2005.0377 PMC1626377

[30] Cingi S, Keinänen M, Vuorinen PJ. Elevated water temperature impairs fertilization and embryonic development of whitefish *Coregonus lavaretus*. J Fish Biol. 2010; 76: 502–521. doi: 10.1111/j.1095-8649.2009.02502.x 20666893 10.1111/j.1095-8649.2009.02502.x

[31] Karnovsky MJ. A formaldehyde-glutaraldehyde fixative of high osmolarity for use in electron microscopy. J Cell Biol. 1963; 27: 137A–138A.

[32] Böck P. Der Semidünnschnitt. Munich: J. F. Bergmann publishers; 1984.

[33] Steinbacher P, Haslett JR, Six M, Gollmann HP, Sänger AM, Stoiber W. Phases of myogenic cell activation and possible role of dermomyotome cells in teleost muscle formation. Dev Dyn. 2006; 235: 3132–3143. doi: 10.1002/dvdy.20950 16960856 10.1002/dvdy.20950

[34] Brack AS, Bildsoe H, Hughes SM. Evidence that satellite cell decrement contributes to preferential decline in nuclear number from large fibres during murine age-related muscle atrophy. J Cell Sci. 2005; 118: 4813–4821. doi: 10.1242/jcs.02602 16219688 10.1242/jcs.02602

[35] Stoiber W, Haslett JR, Wenk R, Steinbacher P, Gollmann HP, Sänger AM. Cellularity changes in developing red and white fish muscle at different temperatures: simulating natural environmental conditions for a temperate freshwater cyprinid. J Exp Biol. 2002; 205: 2349–2364. 12124361 10.1242/jeb.205.16.2349

[36] Steinbacher P, Stadlmayr V, Marschallinger J, Sänger AM, Stoiber W. Lateral fast muscle fibres originate from the posterior lip of the teleost dermomyotome. Dev Dyn. 2008; 237: 3233–3239. doi: 10.1002/dvdy.21745 18924233 10.1002/dvdy.21745 PMC2923027

[37] Marschallinger J, Obermayer A, Sänger AM, Stoiber W, Steinbacher P. A novel mechanism of proliferative Pax7+ cell immigration accounts for postembryonic fast muscle growth in teleost fish. Dev Dyn. 2009; 238: 2442–2448. 19653317 10.1002/dvdy.22049 PMC2923022

[38] Noguchi K, Gel YR, Brunner E, Konietschke F. nparLD: an R software package for the nonparametric analysis of longitudinal data in factorial experiments. J Stat Softw. 2012; 50: 1–23.25317082

[39] Buckingham M, Relaix F. The role of Pax genes in the development of tissues and organs: Pax3 and Pax7 regulate muscle progenitor cell functions. Annu Rev Cell Dev Biol. 2007; 23: 645–673. doi: 10.1146/annurev.cellbio.23.090506.123438 17506689 10.1146/annurev.cellbio.23.090506.123438

[40] Ohlberger J, Brännström Å, Dieckmann U. Adaptive phenotypic diversification along a temperature-depth gradient. Am Nat. 2013; 182: 359–373. doi: 10.1086/671169 23933726 10.1086/671169

[41] Hanel R, Karjalainen J, Wieser W. Growth of swimming muscles and its metabolic cost in larvae of whitefish at different temperatures. J Fish Biol. 1996; 48: 937–951.

[42] Johnston IA, Vieira VLA, Temple G.K. Functional consequences and population differences in the developmental plasticity of muscle to temperature in Atlantic herring *Clupea harengus*. Mar Ecol Prog Ser. 2001; 213: 285–300.

[43] Georga I, Koumoundouros G. Thermally induced plasticity of body shape in adult zebrafish *Danio rerio* (Hamilton, 1822). J Morphol. 2010; 271: 1319–1327. doi: 10.1002/jmor.10874 20715149 10.1002/jmor.10874

[44] Nathanailides C, Lopez-Albors O, Stickland NC. Influence of prehatch temperature on the development of muscle cellularity in posthach Atlantic salmon (*Salmo salar*). Can J Fish Aquat Sci. 1995; 52: 675–680.

[45] Campos C, Valente LM, Conceição LE, Engrola S, Fernandes JM. Temperature affects methylation of the myogenin putative promoter, its expression and muscle cellularity in Senegalese sole larvae. Epigenetics 2013; 8: 389–397. doi: 10.4161/epi.24178 23538611 10.4161/epi.24178 PMC3674048

[46] Fernandes JM, MacKenzie MG, Kinghorn JR, Johnston IA. FoxK1 splice variants show developmental stage-specific plasticity of expression with temperature in the tiger pufferfish. J Exp Biol. 2007; 210: 3461–3472. doi: 10.1242/jeb.009183 17873000 10.1242/jeb.009183

[47] Fuentes EN, Zuloaga R, Valdes JA, Molina A, Alvarez M. Skeletal muscle plasticity induced by seasonal acclimatization involves IGF1 signaling: implications in ribosomal biogenesis and protein synthesis. Comp. Biochem. Physiol. B Biochem. Mol Biol. 2014; 176: 48–57. doi: 10.1016/j.cbpb.2014.07.003 25088252 10.1016/j.cbpb.2014.07.003

