# Supplementary figures and images for: Thermal experience during embryogenesis contributes to the induction of dwarfism in whitefish Coregonus lavaretus
Source: PLoS One. 2017 Sep 25;12(9):e0185384. doi: 10.1371/journal.pone.0185384 (PMC5612755; doi:10.1371/journal.pone.0185384)

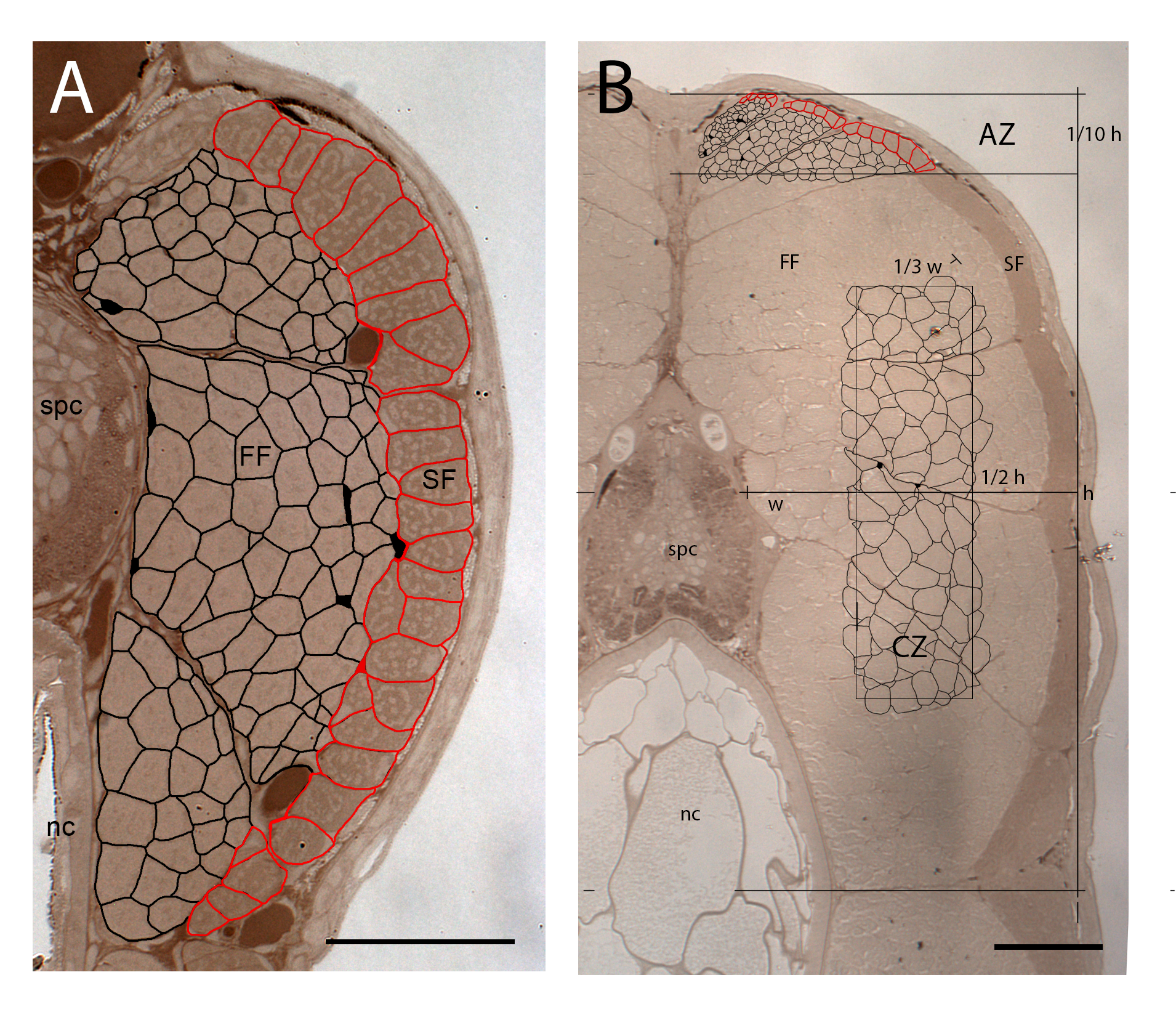

Supplement: S1 Fig — Epaxial quadrants of fish at hatching (A) and at 80 dph (B). Zonal subdivision at 80 dph (AZ apical zone, CZ central zone) is dependent of myotome size (details provided in the Methods section). FF fast fibres, h/w height/width of quadrant, ms myoseptum, nc notochord, SF slow fibres, spc spinal cord. Scale bars: A 50 μm, B 100 μm. (TIF) [file pone.0185384.s001.tif]

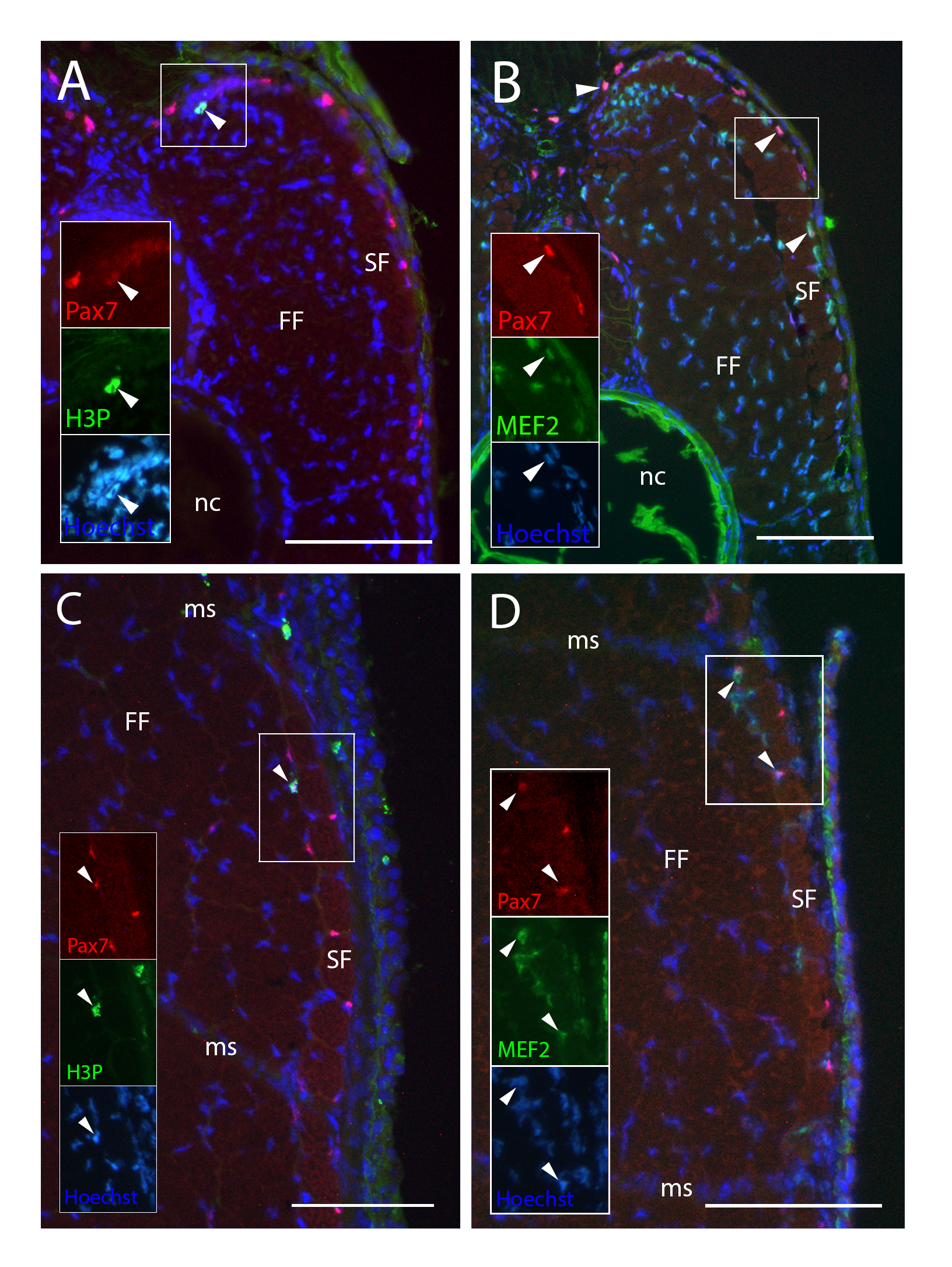

Supplement: S2 Fig — Dorsal is to the top. (A) Newly hatched 2°-fish of dwarf form, section co-stained for H3P (green) to test for mitotically active Pax7+ cells (white arrowhead). All Pax7+ cells are exclusively located in the area of the previous DM. (B) Newly hatched 6°-fish of normal sized-form, section co-stained for MEF2 (green) to test for Pax7+ cells entering myogenic differentiation (white arrowheads). (C) 6°-fish of dwarf form at 80 dph, section co-labelled for H3P (green). Pax7+ cells and mitotically active Pax7+/H3P+ cells generally occur in both the area of the previous DM and in the lateral fast muscle (white arrowhead indicates Pax7+/H3P+ cell). (D) 2°-fish of normal-sized form at 80 dph, section co-stained for MEF2 (green). White arrowheads indicate double-labelled cells. Nuclei are counterstained with Hoechst 33258. FF fast fibres, ms myoseptum, nc notochord, SF slow fibres. Scale bars: 100 μm. (TIF) [file pone.0185384.s002.tif]
